# Brown-Séquard Syndrome Secondary to Cervical Spinal Meningioma: A Case Report

**DOI:** 10.7759/cureus.114884

**Published:** 2026-08-20

**Authors:** Andrew J George, Jimmy Lau, Abdullah Jalal, Joseph Fullmer, Mazen Al-Hakim

**Affiliations:** 1 Neurology, Oakland University William Beaumont School of Medicine, Rochester, USA; 2 Pathology, Corewell Health William Beaumont University Hospital, Royal Oak, USA; 3 Neurology, Corewell Health William Beaumont University Hospital, Royal Oak, USA

**Keywords:** atypical meningioma, brown-séquard syndrome, case report, cervical spine, myelopathy

## Abstract

Brown-Séquard syndrome (BSS) is an incomplete spinal cord injury classically caused by hemisection of the cord, resulting in ipsilateral upper motor neuron weakness and loss of proprioception, vibration, and fine touch, with contralateral loss of pain and temperature sensation. In addition, lower motor neuron signs and segmental sensory deficits may be observed at the level of the lesion. While traumatic etiologies are most commonly reported, compressive lesions such as spinal meningiomas can produce similar clinical presentations. In this case, we present a 66-year-old male with a three-month history of progressive gait difficulty who presented with classic BSS symptoms secondary to a C7 intradural extramedullary meningioma. The patient underwent successful surgical resection via C6-T1 posterior cervical laminectomy with excellent neurological recovery. Histopathological examination revealed a World Health Organization (WHO) Grade 2 atypical meningioma. At the eight-week follow-up, the patient demonstrated significant functional improvement with resolution of spasticity and motor weakness, with only mild residual sensory deficits. This case highlights the importance of recognizing progressive myelopathy symptoms and emphasizes that early surgical intervention for compressive spinal cord lesions can result in excellent functional outcomes, even in elderly patients with atypical meningioma pathology.

## Introduction

Brown-Séquard syndrome (BSS) is an uncommon, incomplete spinal cord injury characterized by ipsilateral weakness and loss of vibration/proprioception with contralateral loss of pain and temperature sensation below the lesion, reflecting the organization of the corticospinal, dorsal column-medial lemniscus, and spinothalamic pathways [1]. Although trauma is a classic cause, BSS may also result from nontraumatic compressive lesions, with cervical disc herniation being the most common surgically treatable etiology. Spinal meningiomas (SMs) have also been reported to present as Brown-Séquard syndrome, highlighting the need for early recognition and urgent imaging [2,3]. Other surgically treatable nontraumatic etiologies include spinal cord herniation, cervical spondylotic stenosis, ossification of the posterior longitudinal ligament, primary or secondary tumors, intramedullary cystic lesions, and selected infectious causes such as Pott's disease and spinal cysticercosis [3].

SMs are the most common primary intradural extramedullary tumors in adults [4] and account for roughly a quarter of primary spinal cord tumors, with a predominance in the thoracic spine followed by the cervical region; SMs typically grow slowly and present with progressive myelopathy [5]. Surgical resection is the mainstay of treatment and, in most series, leads to favorable neurological outcomes [5,6]. While most SMs are World Health Organization (WHO) Grade 1, WHO Grade 2 (atypical) meningiomas constitute a clinically relevant minority with higher risks of recurrence, necessitating closer postoperative magnetic resonance imaging (MRI) surveillance [7,8]. In addition, multiple meningiomas may coexist, which is an important consideration when an incidental second lesion is detected [9].

Here, we report a patient with progressive myelopathy and left Brown-Séquard syndrome due to a C7 intradural extramedullary meningioma, highlight the exam findings that expedited imaging and definitive surgery, and discuss postoperative considerations for WHO Grade 2 pathology and an incidental additional meningioma.

## Case presentation

History and clinical presentation

A 66-year-old male with hypertension, dyslipidemia, and coronary artery disease presented to the neurology clinic with a three-month history of progressive difficulty walking. The patient reported numbness of the right foot and dragging of the left foot when walking. During exercise, the patient experienced pain in both thighs. He also complained of pain between the shoulder blades that had developed over the same time period. He most recently developed urinary urgency without incontinence. There was no family history of central nervous system tumors or neurofibromatosis.

The patient noted a mild worsening of symptoms over the four days prior to his neurology consultation, which he attributed to beginning physical therapy during that time. His symptoms gradually progressed over the three-month period without any acute changes or precipitating events.

Physical examination

Neurologic examination demonstrated findings consistent with progressive myelopathy, including left lower-extremity spasticity with bilateral hip flexor weakness and left ankle dorsiflexion weakness. Deep tendon reflexes were brisk at the knees bilaterally with crossed adductor responses. Upper motor neuron signs were present, including bilateral Babinski and Chaddock responses.

Sensory examination revealed impaired vibration bilaterally, extending up to the ankle on the right side and up to the shin on the left side. Impaired pinprick sensation was present in the right leg extending all the way up to the T9 dermatome level. Gait examination revealed an abnormal pattern with dragging of the left leg.

The clinical picture was consistent with progressive myelopathy with a left BSS pattern. Given the constellation of findings and concern for a space-occupying lesion in the spine, the patient was advised to proceed immediately to the emergency department for urgent imaging evaluation.

Imaging studies

**Figure 1:**
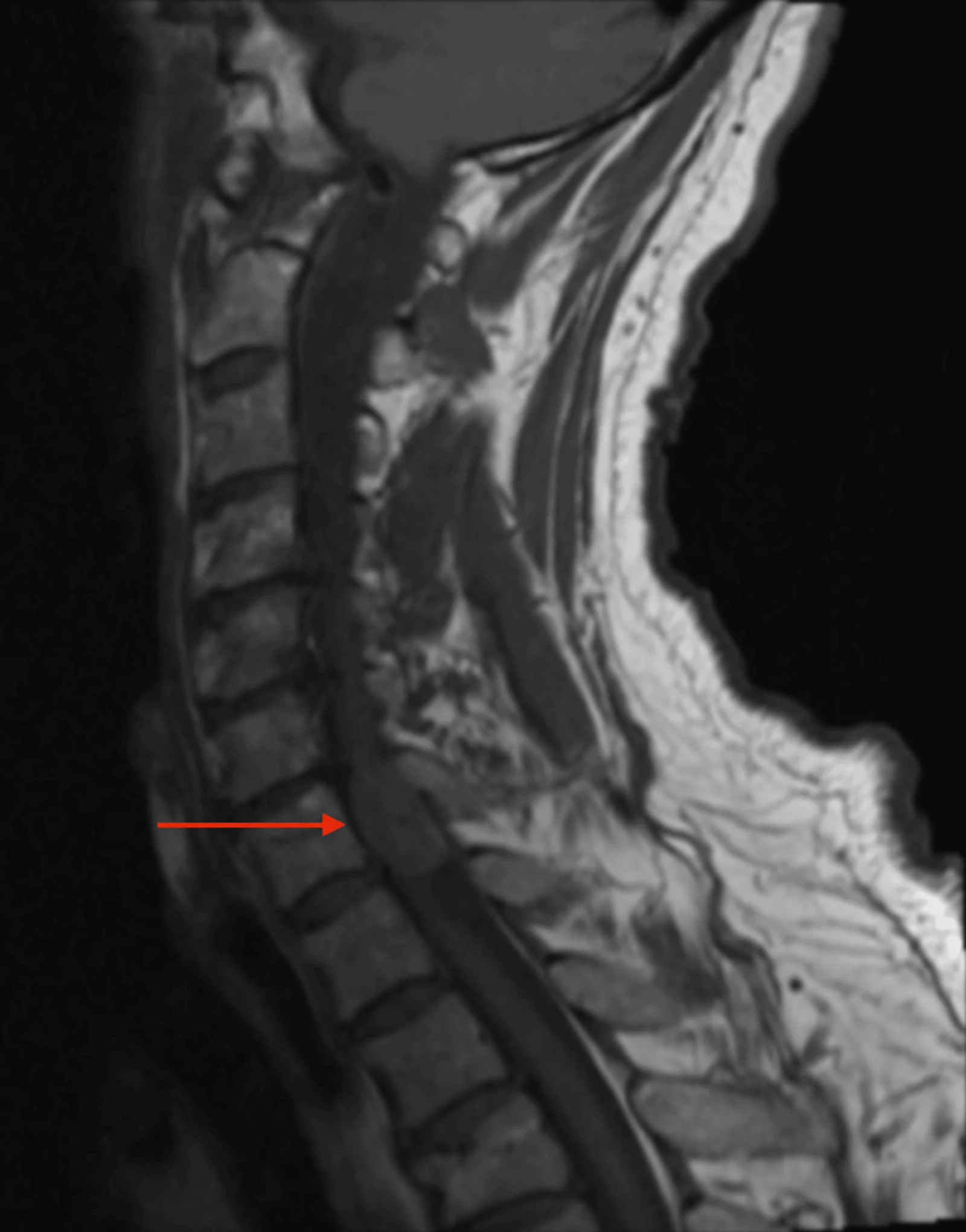
T1 without contrast spinal MRI Sagittal T1-weighted cervical spine MRI without contrast demonstrating an enhancing intradural extramedullary mass consistent with a meningioma, producing focal spinal cord compression.

**Figure 2:**
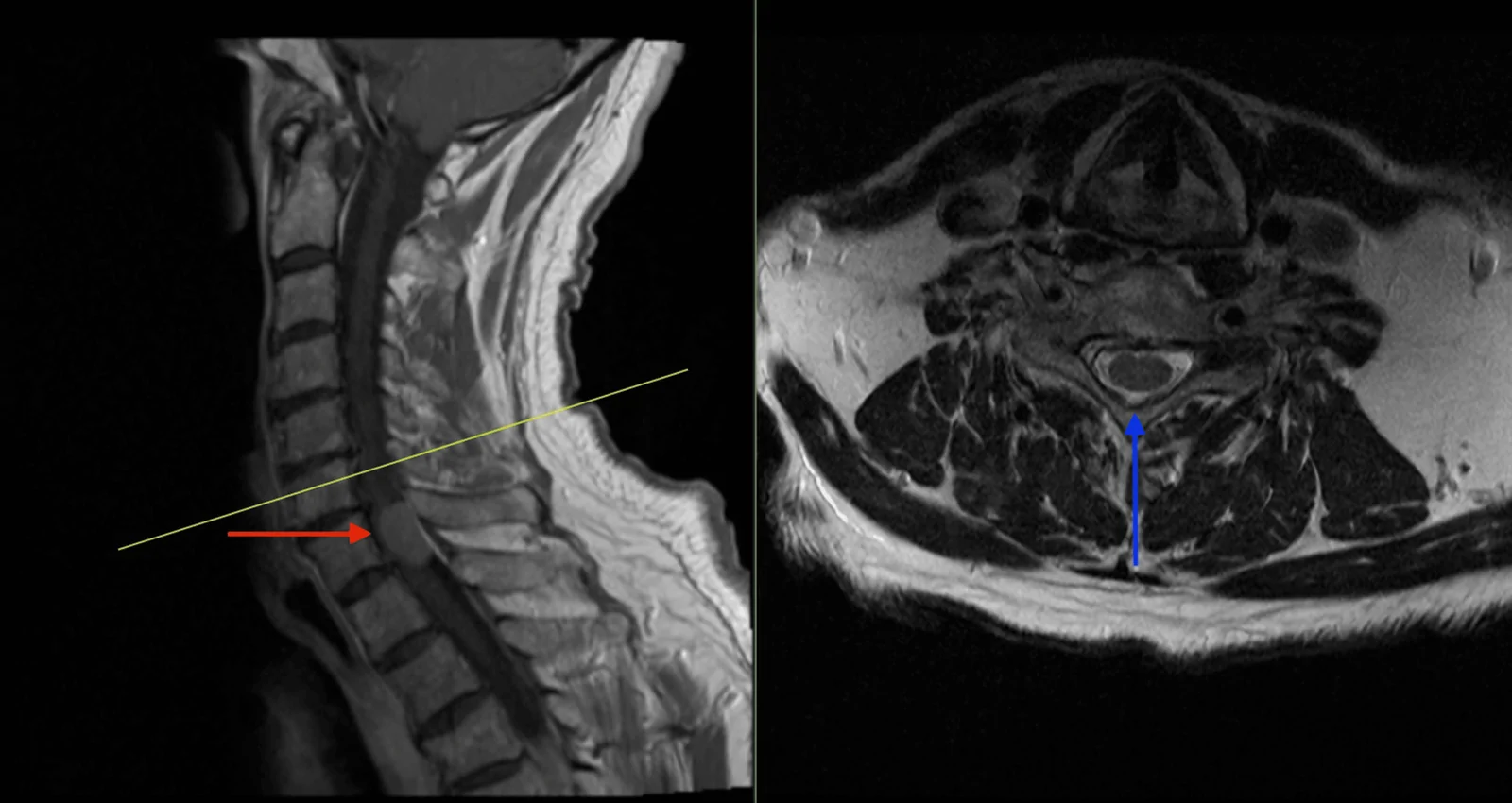
T1 with contrast spinal MRI without meningioma in axial view Sagittal (left) and axial (right) T1-weighted cervical spine MRI with contrast demonstrating normal soft-tissue and spinal canal anatomy without evidence of a meningioma. The sagittal slice plane is indicated by the yellow line.

**Figure 3:**
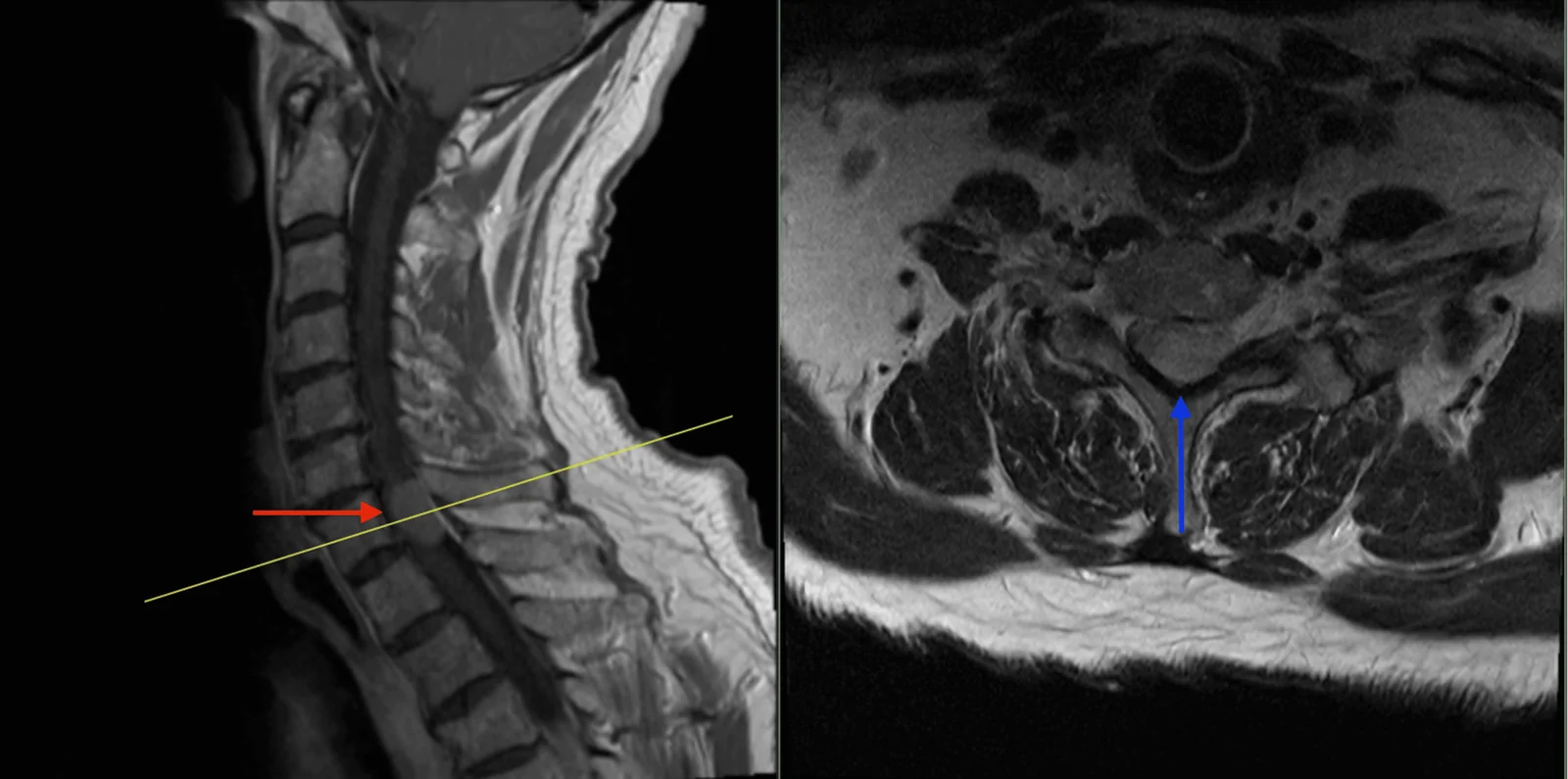
T1 with contrast spinal MRI with meningioma in axial view Sagittal (left) and axial (right) T1-weighted cervical spine MRI with contrast demonstrating an intradural extramedullary mass on the axial view, consistent with a meningioma, causing displacement of the cervical spinal cord. The sagittal slice position is indicated by the yellow line.

Magnetic resonance imaging of the cervical spine revealed a 2 cm intradural extramedullary mass at the C7 level, with imaging characteristics consistent with meningioma (Figures 1-3). The lesion demonstrated significant mass effect with ventral and rightward displacement of the spinal cord. Additionally, there was a stress appearance of the exiting C8 nerve root secondary to the mass effect. No pathological enhancement of the spinal cord or the spinal canal itself was observed.

Interestingly, postoperative brain magnetic resonance imaging revealed an incidental 25 × 8.5 mm dural-based enhancing extra-axial lesion in the middle cranial fossa along the left temporal convexity, which was also consistent with meningioma. This finding was not addressed in subsequent neurosurgical visits, suggesting a conservative approach given its asymptomatic nature.

Surgical intervention

Nine days after the initial neurology consultation, the patient underwent C6-T1 posterior cervical laminectomy with resection of the intradural extramedullary meningioma. The surgical procedure was performed without complications, and the tumor was completely resected.

Histopathological analysis

**Figure 4:**
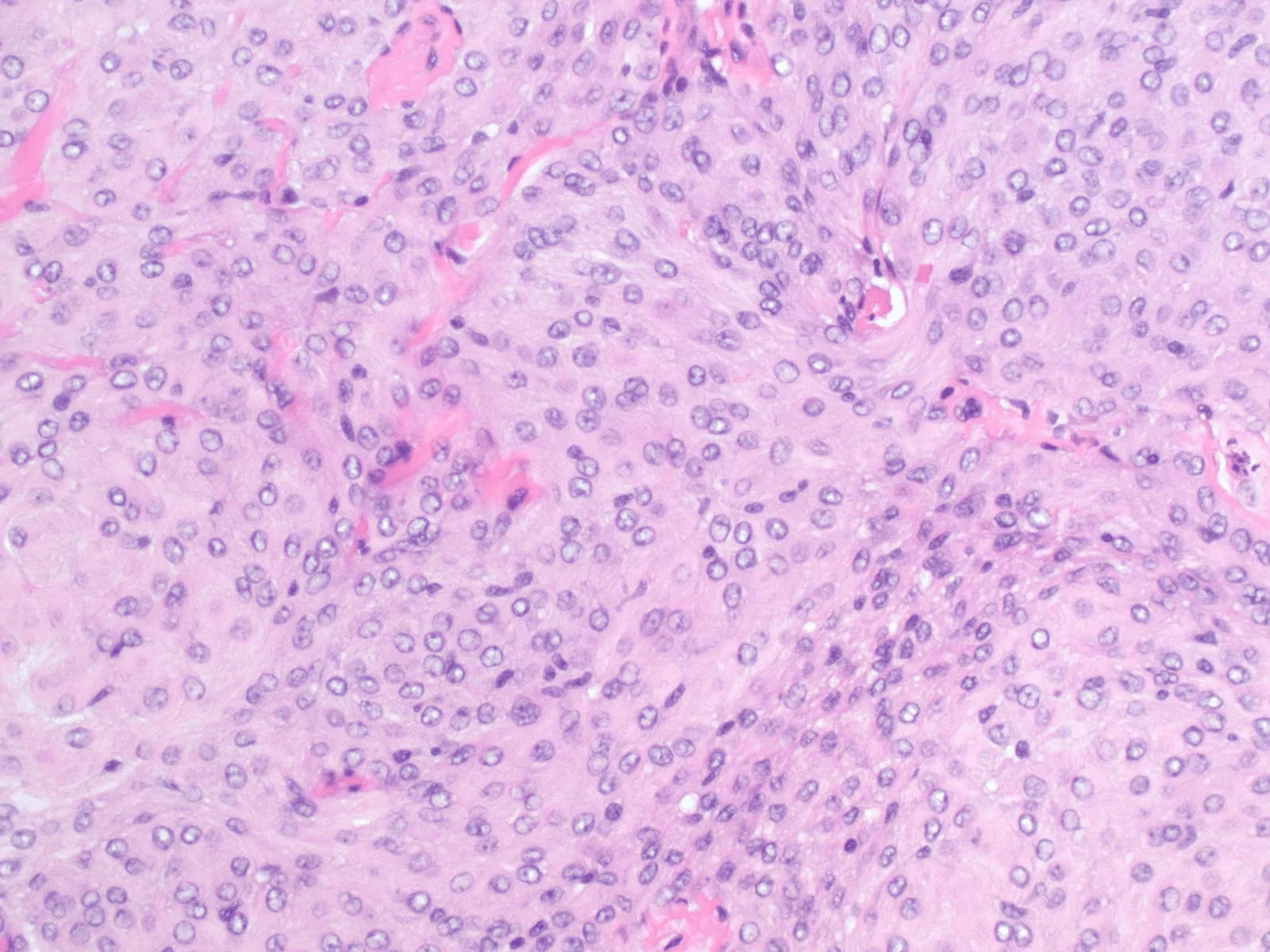
Representative H&E-stained section highlighting area of prominent nucleoli

**Figure 5:**
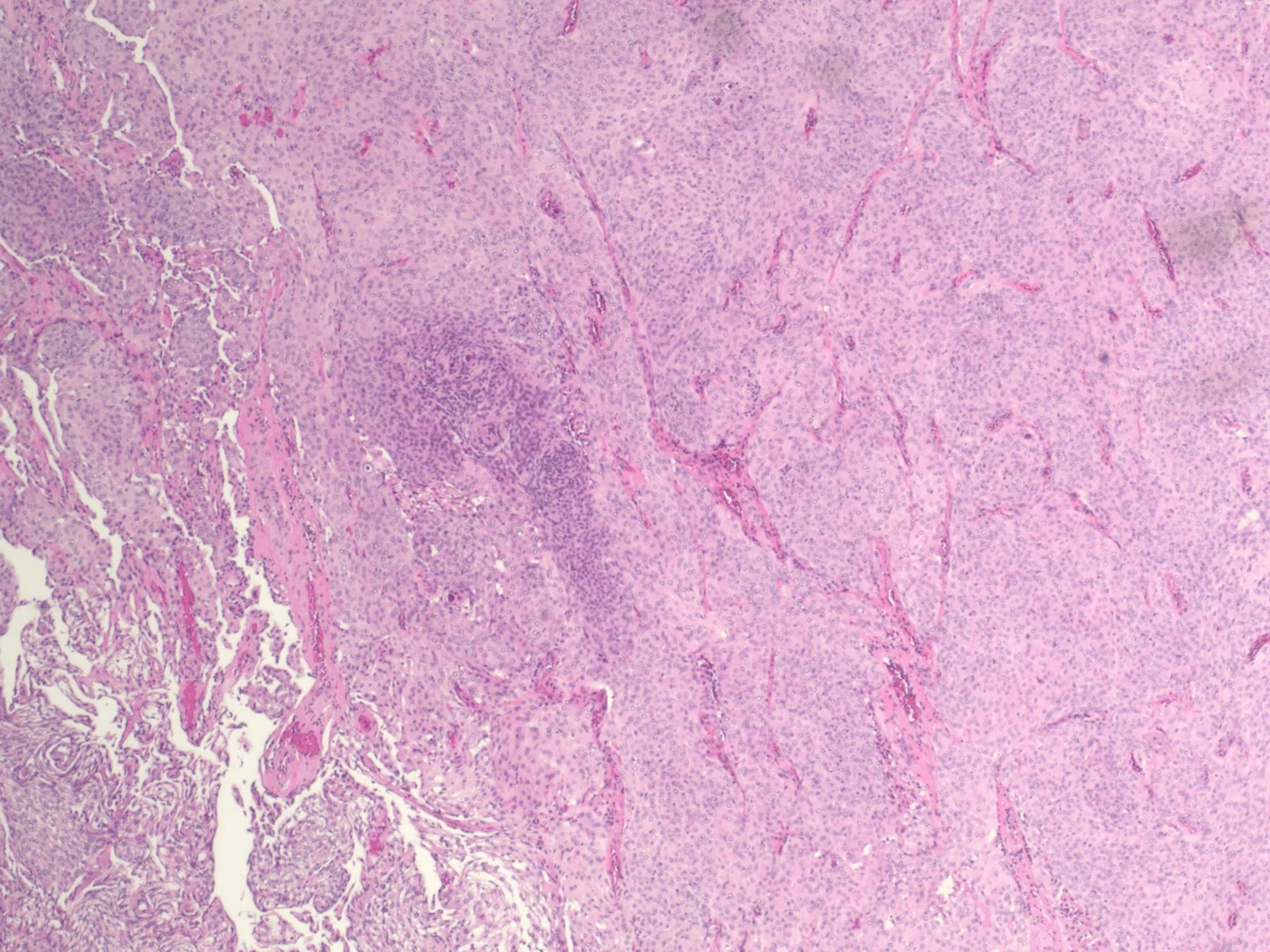
Representative H&E-stained section highlighting small cells with elevated nuclear/cytoplasmic ratio, high cellularity and focal sheeting (4/5 minor criteria are present therefore criteria for atypical meningioma are met. Geographic necrosis is not identified. Increased (4 or more) mitotic activity was not identified.)

Pathological examination of the resected specimen revealed atypical WHO Grade 2 meningioma (Figures 4, 5). This finding has implications for long-term prognosis, as for WHO Grade 2 meningiomas, recurrence rates of ~31% have been reported even after Simpson I-II resection in one surgical series [7]. Accordingly, the European Association of Neuro-Oncology recommends closer postoperative MRI surveillance in patients with higher-grade meningiomas [8].

Clinical outcomes

At the first follow-up visit (13 days postoperatively), the patient demonstrated marked neurological improvement. There was no gait spasticity, left hip flexion strength had returned to baseline, and the patient experienced only mild residual numbness in the right foot. The patient was cleared for physical therapy to begin two weeks following this visit. At the eight-week follow-up visit, no acute neurological issues or deficits were observed. The patient was then able to initiate physical therapy for continued rehabilitation and functional optimization.

## Discussion

This case demonstrates a classic presentation of BSS secondary to compressive myelopathy from a cervical spinal meningioma. The patient's clinical presentation demonstrated the classic neuroanatomical pattern: ipsilateral motor weakness and loss of dorsal column modalities with contralateral pain/temperature loss below the lesion [1]. The gold standard imaging modality for spinal meningiomas is MRI, which then allows for surgical planning if permitted; however, WHO classification is based on histopathological findings [10]. WHO grade 2 atypical meningioma criteria include increased mitotic activity, brain invasion, clear cell or chordoid histology, or at least three of five atypical histologic features, including increased cellularity, small cells with a high nuclear-to-cytoplasmic ratio, prominent nucleoli, sheet-like growth, and spontaneous necrosis [10]. In this case, the histopathologic features supporting WHO grade 2 classification are shown in Figures 4-5. Although molecular alterations such as TERT promoter mutation and CDKN2A/B homozygous deletion are increasingly recognized in meningioma grading and prognostication, molecular testing was not included in the available clinical workup; therefore, classification in this case was based on the reported histopathologic criteria [10].

The WHO Grade 2 pathology adds complexity to long-term management, given higher recurrence/progression risks than grade 1 tumors do; thus, MRI surveillance at shorter intervals is generally recommended after resection of Grade 2 meningioma [7,8]. The incidental discovery of a second intracranial meningioma underscores that multiple meningiomas are not rare when systematically sought. Contemporary series report higher rates than historical estimates do, supporting the need for tailored but comprehensive imaging and long-term surveillance [9]. The coexistence of spinal and intracranial meningiomas, as observed in our patient, has been reported in the literature, especially in the context of multiple meningiomas [4].

Although BSS is a rare neurological condition, it can severely disrupt independence in daily life, particularly through impairments in mobility, balance, and sensory perception. While most cases are due to trauma and present acutely, our patient did not seek medical care until the symptoms became progressively worse over several months. Our case underscores the need for clinicians to remain vigilant and maintain a broad differential diagnosis for patients presenting with hemicord neurologic symptoms, including compressive neoplasms such as meningioma, infectious etiologies such as Pott’s disease, inflammatory/demyelinating disease, vascular causes, and other structural lesions. Early diagnosis of BSS with an etiology due to a neoplastic lesion of the spinal cord and early intervention through resection can prevent irreversible spinal cord damage. Through patient education, the public can learn to recognize certain neurological signs that warrant urgent medical care.

## Conclusions

This case report illustrates the classic presentation of BSS secondary to cervical spinal meningioma and demonstrates the excellent outcomes possible with prompt recognition and surgical intervention. Key learning points include the importance of recognizing progressive myelopathy symptoms on physical examination, understanding the neuroanatomical basis of BSS, and the potential for excellent functional recovery following surgical decompression of compressive spinal cord lesions. WHO Grade 2 pathology necessitates long-term surveillance given increased recurrence risks, and additional asymptomatic meningiomas may coexist.
